# Transcriptome integration analysis and specific diagnosis model construction for Hodgkin's lymphoma, diffuse large B-cell lymphoma, and mantle cell lymphoma

**DOI:** 10.18632/aging.202882

**Published:** 2021-04-22

**Authors:** Wen-Xing Li, Shao-Xing Dai, San-Qi An, Tingting Sun, Justin Liu, Jun Wang, Leyna G. Liu, Yang Xun, Hua Yang, Li-Xia Fan, Xiao-Li Zhang, Wan-Qin Liao, Hua You, Luca Tamagnone, Fang Liu, Jing-Fei Huang, Dahai Liu

**Affiliations:** 1 Department of Biochemistry and Molecular Biology, School of Basic Medical Sciences, Southern Medical University, Guangzhou, Guangdong, China; 2 Guangdong Provincial Key Laboratory of Single Cell Technology and Application, Southern Medical University, Guangzhou, Guangdong, China; 3 Yunnan Key Laboratory of Primate Biomedical Research, Institute of Primate Translational Medicine, Kunming University of Science and Technology, Kunming, Yunnan, China; 4 Biosafety Level-3 Laboratory, Life Sciences Institute & Guangxi Key Laboratory of AIDS Prevention and Treatment & Guangxi Collaborative Innovation Center for Biomedicine, Guangxi Medical University, Nanning, Guangxi, China; 5 National School of Development, Peking University, Beijing 100871, China; 6 Department of Statistics, University of California, Riverside, CA 92521, USA; 7 Foshan Stomatology Hospital, School of Medicine, Foshan University, Foshan, Guangdong, China; 8 Portola High School, Irvine, CA 92618, USA; 9 Affiliated Cancer Hospital & Institute of Guangzhou Medical University, Guangzhou, Guangdong, China; 10 Istituto di Istologia ed Embriologia, Università Cattolica del Sacro Cuore, Rome, Italy; 11 Key Laboratory of Animal Models and Human Disease Mechanisms, Kunming Institute of Zoology, Chinese Academy of Sciences, Kunming, Yunnan, China

**Keywords:** lymphoma, gene expression, diagnostic model, marker gene, intergroup difference

## Abstract

Transcriptome differences between Hodgkin's lymphoma (HL), diffuse large B-cell lymphoma (DLBCL), and mantle cell lymphoma (MCL), which are all derived from B cell, remained unclear. This study aimed to construct lymphoma-specific diagnostic models by screening lymphoma marker genes. Transcriptome data of HL, DLBCL, and MCL were obtained from public databases. Lymphoma marker genes were screened by comparing cases and controls as well as the intergroup differences among lymphomas. A total of 9 HL marker genes, 7 DLBCL marker genes, and 4 MCL marker genes were screened in this study. Most HL marker genes were upregulated, whereas DLBCL and MCL marker genes were downregulated compared to controls. The optimal HL-specific diagnostic model contains one marker gene (MYH2) with an AUC of 0.901. The optimal DLBCL-specific diagnostic model contains 7 marker genes (LIPF, CCDC144B, PRO2964, PHF1, SFTPA2, NTS, and HP) with an AUC of 0.951. The optimal MCL-specific diagnostic model contains 3 marker genes (IGLV3-19, IGKV4-1, and PRB3) with an AUC of 0.843. The present study reveals the transcriptome data-based differences between HL, DLBCL, and MCL, when combined with other clinical markers, may help the clinical diagnosis and prognosis.

## INTRODUCTION

Lymphoma is a malignant tumor originating from the lymphoid hematopoietic system and is mainly divided into two categories: Hodgkin's lymphoma (HL) and non-Hodgkin's lymphoma (NHL). Lymphoma is considered to be a chemosensitive tumor and the risk of lymphoma increases significantly with age [1]. Weakened organismal functions, defects in cellular and tissue homeostasis, immune deficiency, and multiple genetic alterations such as increased DNA damage in cells were correlated with aging. These risk factors are also the main causes of many cancers such as lymphoma [2]. Studies have reported that many anti-aging measures are also helpful in the treatment of lymphoma [2]. Enhancing the expression of anti-aging genes can be an effective way to inhibit lymphoma, a recent study showed that enforced expression of Klotho could significantly induce cell apoptosis and inhibit tumor growth in diffuse large B-cell lymphoma (DLBCL) [3]. Furthermore, downregulated telomere-binding genes (TRF1, TRF2, and POT1) lead to complex chromosomal aberrations, alternative lengthening of telomeres, and induced the progression of HL [4].

According to the WHO classification of lymphoid neoplasias (2016 version), more than 40 types of lymphoma are recognized, with clinical behaviors spanning from remarkably indolent to profoundly aggressive [5]. There are many subtypes of NHL and the most common of which is DLBCL. HL and DLBCL are B-cell-derived lymphomas with high incidence [6]. Mantle cell lymphoma (MCL) is a rare type of B-cell lymphoma that is still incurable, accounting for about 3-6% of all NHL because of its high malignant aggressiveness [7]. There are large differences in histological classification, pathological diagnostic markers, clinical treatment, and prognostic status among HL, DLBCL, and MCL [7–9]. The prognosis of the three B cell-derived lymphoma subtypes is quite different [7, 9, 10]. Especially the prognosis of MCL patients is very poor, and many therapy methods have not achieved the expected outcomes [11].

The different types of lymphoma or lymphoma subtypes can be distinguished by gene expression profiling [12]. Several genes can be used as diagnostic markers for specific types of lymphoma. The ligands of the tumor necrosis factor (TNF) family (APRIL and BAFF) showed high specificity and sensitivity in the diagnosis of central nervous system lymphoma [13]. The high expression of FOXP-1 in pediatric-type follicular lymphoma can also distinguish it from follicular hyperplasia [14]. Furthermore, high-throughput T cell receptor (TCR) gene sequencing technology facilitates the detection of early-stage cutaneous T-cell lymphoma [15]. However, most of the previous lymphoma diagnostic or prognostic models did not consider the heterogeneity among different tumors or subtypes [16–20]. Due to a large number of lymphoma subtypes, some genes may show consistent differential expression in multiple lymphoma subtypes that may interfere with the diagnostic specificity. Furthermore, complex gene-gene interactions also affect the accuracy of tumor diagnosis [21] and prognostic status [22]. Therefore, a diagnostic model with excellent performance should also be robust and hardly affected by gene-gene interactions.

The identification of tumor subtypes contributes to the effective treatment of the disease and prolongs the survival of cancer patients. Using gene expression characteristics to screen specific molecular markers is an effective approach to distinguish tumor subtypes. Through the identification of subtype-specific genes and constructing corresponding models, researchers can accurately perform subtype-specific diagnosis and prognostic evaluation for various tumor patients [23–25]. Currently, the diagnosis of HL, DLBCL, and MCL is mainly based on the morphology and the different combinations of CD surface antigens [26]. The clinical application of genotyping differences among these lymphomas is still limited, and there is lacking an effective molecular diagnostic model. Therefore, this study aims to screen for subtype-specific marker genes and constructed lymphoma-specific diagnostic models, and then explore the related biological functions and prognostic status of these specific molecular markers.

## RESULTS

### Differentially expressed genes overview

**Figure 1:**
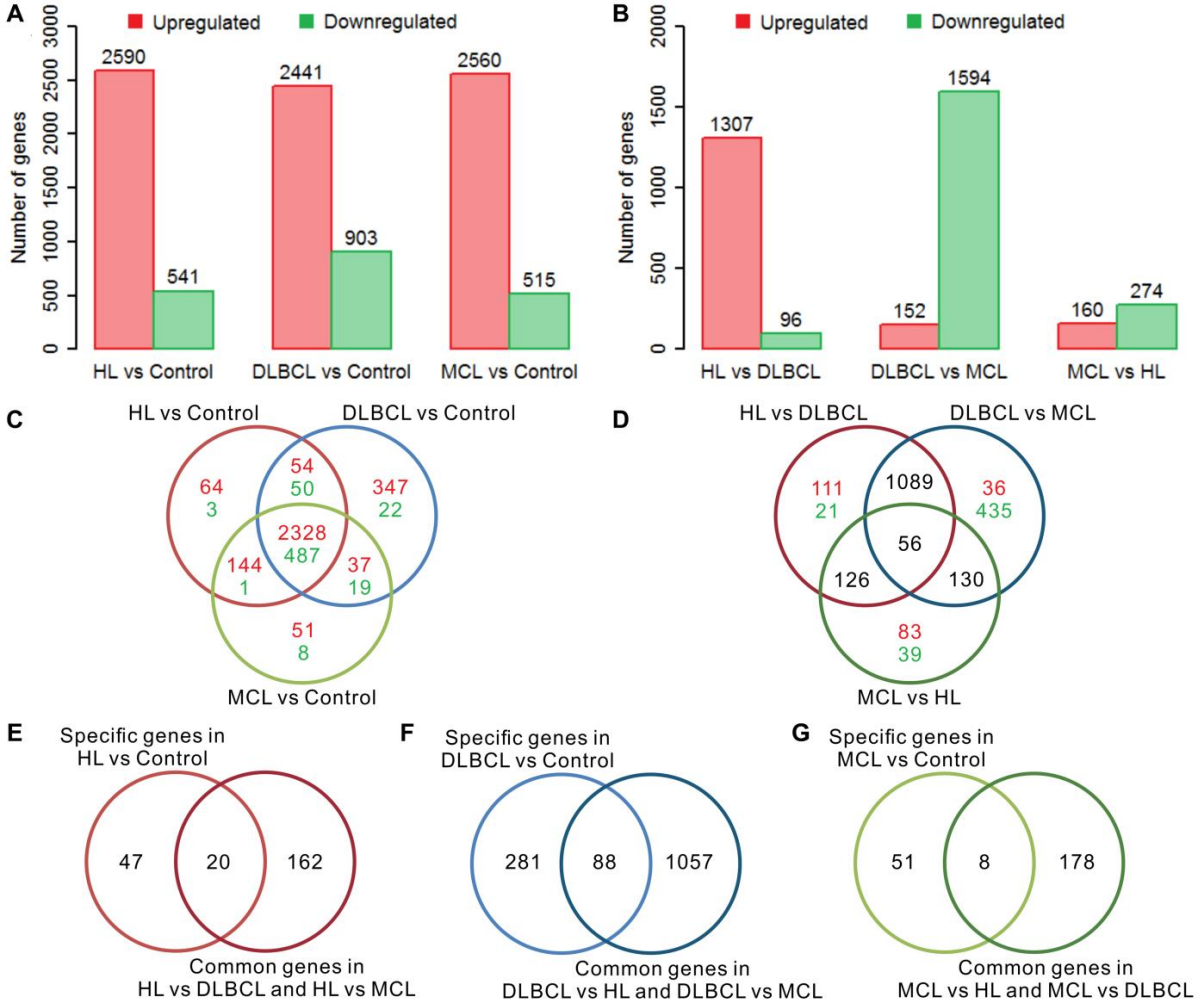
**Differential gene expression analysis of three lymphomas.** (**A**) The number of differentially expressed genes (DEGs) in lymphoma samples compared to controls. (**B**) The number of intergroup difference genes (IDGs) in three types of lymphoma. (**C**) Venn diagram of DEGs in lymphomas compared to controls. The red color indicates the number of upregulated genes and the green color indicates the number of downregulated genes. The expression trends of these genes are consistent in different types of lymphoma compared with controls. (**D**) Venn diagram of the IDGs between the lymphoma groups. The red color indicates the number of upregulated genes and the green color indicates the number of downregulated genes. (**E**) Venn diagram of HL-specific DEGs and HL common IDGs. (**F**) Venn diagram of DLBCL-specific DEGs and DLBCL common IDGs. (**G**) Venn diagram of MCL-specific DEGs and MCL common IDGs. The red bar indicates the upregulated genes, and the green bar indicates the downregulated genes. HL, Hodgkin's lymphoma; DLBCL, diffuse large B-cell lymphoma; MCL, mantle cell lymphoma.

There were more than 3000 differentially expressed genes (DEGs) in the tumor samples compared to the controls in each type of lymphoma, and most of the genes were upregulated (Figure 1A). Most of these differentially expressed genes are upregulated, and a few genes are downregulated. This result is consistent with previous reports that the number of up-regulated genes in different lymphomas is much greater than down-regulated genes [27–29]. Regarding the intergroup comparisons, DLBCL showed a large difference compared with the other two lymphomas, whereas only a small difference was detected between HL and MCL (Figure 1B). There were 67, 369, and 59 specific DEGs in HL vs. control, DLBCL vs. control, and MCL vs. control, respectively (Figure 1C). Furthermore, the results of the intergroup comparisons suggest that there were 182 intergroup difference genes (IDGs) shared by HL vs. DLBCL and HL vs. MCL, 1145 IDGs shared by DLBCL vs. HL and DLBCL vs. MCL, and 186 IDGs shared by MCL vs. HL and MCL vs. DLBCL (Figure 1D). According to the screening criteria for lymphoma-specific genes (defined as the intersection of specific differentially expressed genes in lymphoma samples compared to controls and the common intergroup differentially expressed genes in different lymphoma groups), we identified 20 HL-specific genes (Figure 1E), 88 DLBCL-specific genes (Figure 1F) and 8 MCL-specific genes (Figure 1G). The GO enrichment results showed that the HL specific genes are mainly involved in muscle functions, differentiation, and development functions; the DLBCL specific genes are mainly involved in proliferation, development, and neuromodulation functions; and the MCL specific genes are mainly involved in multiple immune-related functions (Supplementary Table 1). Interestingly, most of the HL-specific genes were upregulated, whereas more than 90% of the DLBCL-specific genes were downregulated. Half of the MCL-specific genes were upregulated while the other half were downregulated.

### Expression and function of the lymphoma marker genes

**Figure 2:**
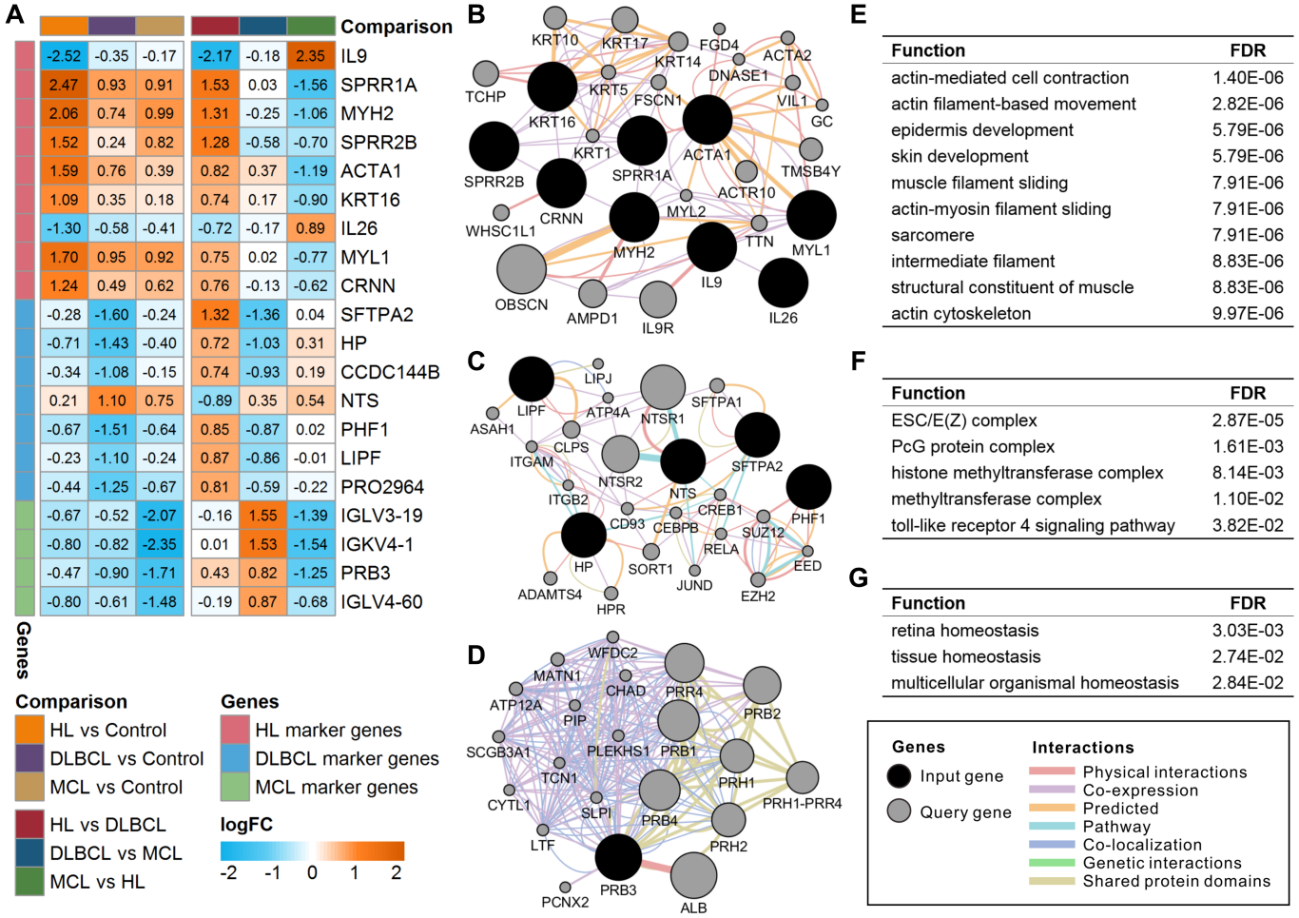
**Expression and functional interaction network of lymphoma marker genes.** (**A**) Log2 transformed the fold-change (logFC) of lymphoma marker genes in different comparisons. The orange color indicates the logFC of the gene > 0, and the cyan color indicates the logFC of the gene < 0. (**B**) Functional interaction network of HL marker genes. (**C**) Functional interaction network of DLBCL marker genes. No records of CCDC144B or PRO2964 were found in the GeneMANIA database. (**D**) Functional interaction network of MCL marker genes. No records of IGLV3-19, IGKV4-1, or IGLV4-60 were found in the GeneMANIA database. (**E**) Enriched functions of HL marker genes and query genes. (**F**) Enriched functions of DLBCL marker genes and query genes. (**G**) Enriched functions of MCL marker genes and query genes. HL, Hodgkin's lymphoma; DLBCL, diffuse large B-cell lymphoma; MCL, mantle cell lymphoma.

There were 20 lymphoma-specific genes (9 HL marker genes, 7 DLBCL marker genes, and 4 MCL marker genes) with a mean absolute value of intergroup fold-change high than 0.5 that were defined as lymphoma marker genes (Figure 2A). Among these genes, IL9, SFTPA2, and IGLV3-19 showed the highest specificity in HL, DLBCL, and MCL, respectively. The GO enrichment results showed that these marker genes were mainly involved in the regulation of various immune response and metabolic processes (Supplementary Table 2). Gene-gene interaction analysis proved that most marker genes were independently correlated with lymphoma status (Supplementary Table 3). The high expression of MYH2 increased HL risk whereas the high expression of LIPF and IGLV3-19 reduced DLBCL and MCL risk. The functional interaction network shows that most of the HL marker genes showed coexpression relationships with each other (Figure 2B). DLBCL and MCL marker genes showed multiple interaction relationships with other genes (Figure 2C and 2D). The enrichment results suggest that HL marker genes are mainly involved in actin- and cytoskeleton-related functions (Figure 2E), DLBCL marker genes are mainly involved in chromatin modification and regulation processes (Figure 2F), and MCL marker genes correlate with organismal homeostasis (Figure 2G). The prognostic analysis shows that IL9 and CRNN correlated with the International Prognostic Score (IPS) in HL (Supplementary Figure 1). Furthermore, low expression of CCDC144B and PHF1 and high expression of HP, LIPF, and SFTPA2 correlate with poor overall survival and progression-free survival in DLBCL (Supplementary Figure 2).

### Single-gene prediction model

**Figure 3:**
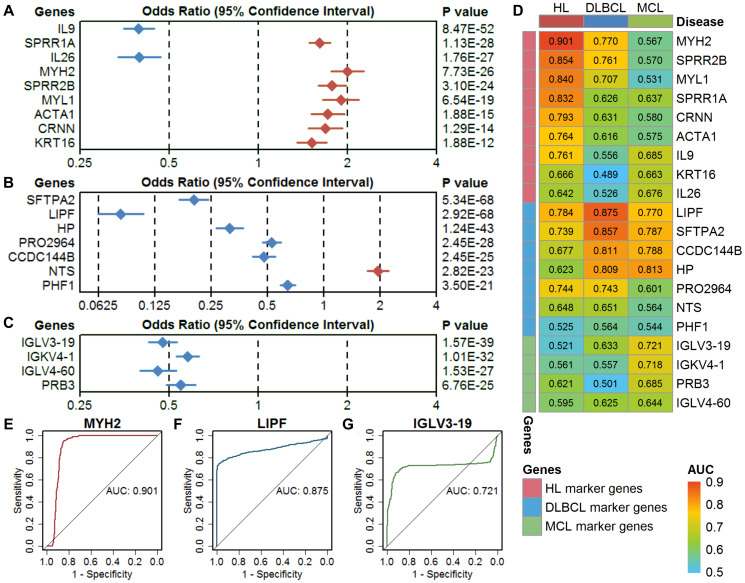
**Evaluation of single-gene models in three types of lymphoma.** (**A**–**C**) The classification performance of HL marker genes, DLBCL marker genes, and MCL marker genes using a univariate logistic regression model. The diamond shape indicates the odds ratio (OR), and the line indicates the 95% confidence interval (CI). The red color indicates OR > 1, and the blue color indicates OR < 1. (**D**) The area under the curve (AUC) of the marker genes in three types of lymphoma. (**E**–**G**) Receiver operating characteristic (ROC) curves of the optimal single-gene model in HL (MYH2), DLBCL (LIPF), and MCL (IGLV3-19). HL, Hodgkin's lymphoma; DLBCL, diffuse large B-cell lymphoma; MCL, mantle cell lymphoma.

A logistic regression model showed that all these marker genes could significantly separate the lymphoma samples from the controls (Figure 3A–3C). The odds ratios of these marker genes are relatively consistent with the expression difference between lymphomas and controls. The results of the ROC analysis of these marker genes are shown in Figure 3D. The ideal classification effect of marker genes is that they have high sensitivity and specificity in the specific type of lymphoma (the AUC value is close to 1) and have a random effect for the other two lymphomas (the AUC value is close to 0.5). For the HL marker genes, MYH2 showed the highest AUC of 0.901 in HL, a low AUC in DLBCL, and an AUC close to 0.5 in MCL; therefore, it can be used as the optimal model in the single-gene prediction model in HL (Figure 3E). LIPF showed the highest AUC of 0.875 in DLBCL and low AUCs in HL and MCL and is considered to be the optimal single-gene prediction model in DLBCL (Figure 3F). However, IGLV3-19 had the highest AUC in MCL marker genes but only showed a general prediction effect (Figure 3G).

### Multigene prediction model

**Figure 4:**
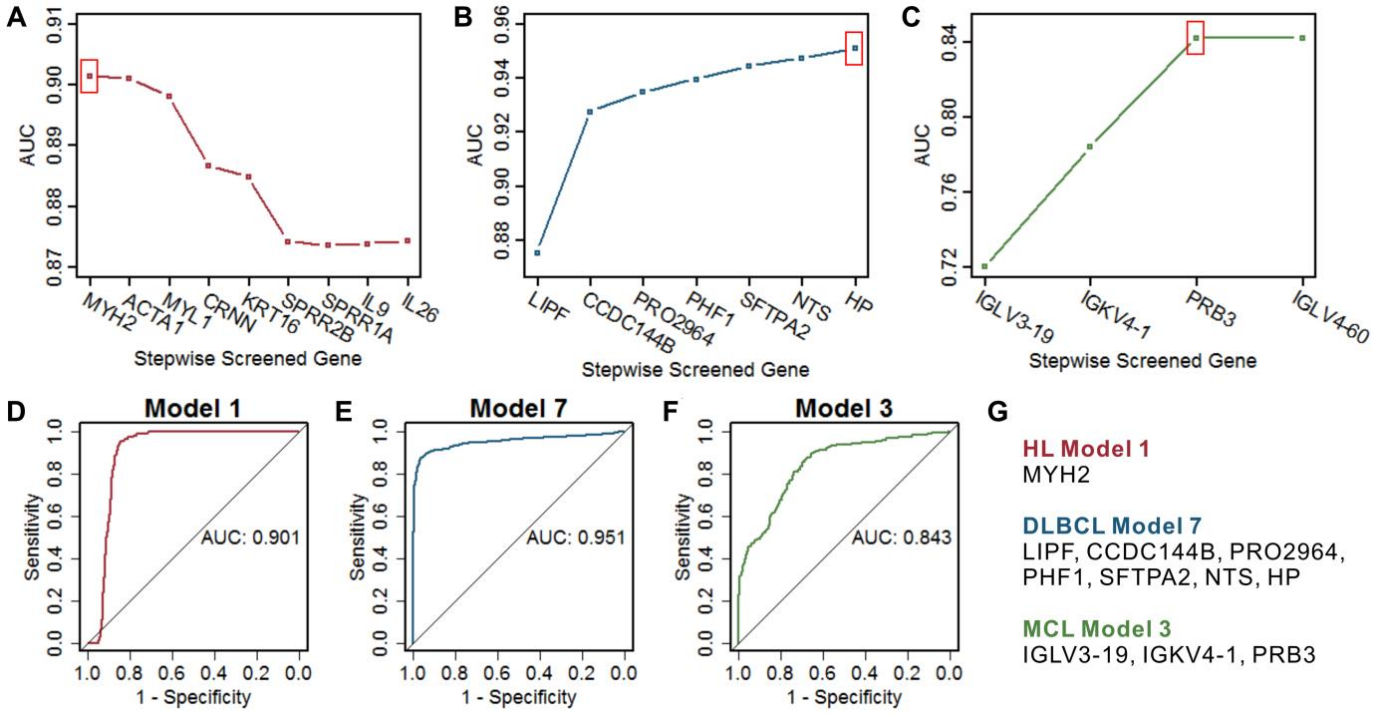
**Screening of the optimal multigene prediction model for three lymphomas.** (**A**–**C**) Stepwise screened multigene prediction models in HL, DLBCL, and MCL. From left to right on the x-axis (stepwise screened genes), each additional gene corresponds to a model [for example, in (A), MYH2 represents model 1, which contains one gene of MYH2, ACTA1 represents model 2, which contains two genes including MYH2 and ACTA1]. The red box shows the optimal model for each type of lymphoma. (**D**–**F**) ROC curves of the screened optimal models for each type of lymphoma. (**G**) Genes in the screened optimal models for three lymphomas. HL, Hodgkin's lymphoma; DLBCL, diffuse large B-cell lymphoma; MCL, mantle cell lymphoma.

The optimal model in HL is MYH2 (Figure 4A and 4G), which had the highest AUC compared with the remaining gene combination models (Figure 4D). The optimal model in DLBCL is the combination of 7 marker genes, including LIPF, CCDC144B, PRO2964, PHF1, SFTPA2, NTS, and HP (Figure 4B and 4G), which had the highest AUC of 0.951 (Figure 4E). The optimal model in MCL is the combination of 3 marker genes, including IGLV3-19, IGKV4-1, and PRB3 (Figure 4C and 4G), which had the highest AUC of 0.843 (Figure 4F). These three optimal models all show high specificity in a certain type of lymphoma and showed relatively poor specificity for the other two types of lymphomas (Supplementary Figure 3). Considering the analyzed gene expression data derived from samples including not only lymphoma cells, but also stroma. We screened the data derived from isolated lymphoma cells and normal B cells (Table 1) and analyzed the expression of the lymphoma marker genes between cases and controls. Despite the small sample size, most marker genes still showed the differential expression consistent with the overall analysis (Supplementary Figure 4). Furthermore, the optimal diagnostic models of these genes showed high prediction accuracy in the data derived from isolated lymphoma cells (Supplementary Figure 5). The dataset of GSE132929 including multiple types of lymphomas (no HL or controls) was used to verify the predictive performance of the above optimal models. Previous studies suggested that it is difficult to distinguish Burkitt's lymphoma (BURL) and DLBCL [30, 31], the DLBCL optimal model showed a high AUC of 0.843 in the validation set with removed BURK (Supplementary Figure 6).

**Table 1:** Information on the datasets of three types of lymphoma.

**GEO ID**	**Contributor**	**Samples**	**Sample type**	**Platform**
***Hodgkin's lymphoma (HL)***				
GSE7788^1^	Van Loo P, 2007	10 cases 1 control	lymph nodes	Affymetrix HG-U133 Plus 2.0 Array (GPL570)
GSE12453^2^	Brune V, 2008	17 cases 25 controls	isolated lymphoma cells (case) isolated normal B cells (control)	Affymetrix HG-U133 Plus 2.0 Array (GPL570)
GSE13996	Chetaille B. 2008	64 cases	lymph nodes	Affymetrix HG-U133A 2.0 Array (GPL571)
GSE17920	Steidl C, 2009	130 cases	lymph nodes	Affymetrix HG-U133 Plus 2.0 Array (GPL570)
GSE47044	Hartmann S, 2013	19 cases 5 controls	isolated lymphoma cells (case) isolated normal B cells (control)	Affymetrix Human Gene 1.0 ST Array (GPL6244)
***Diffuse large B-cell lymphoma (DLBCL**)*				
GSE12453^2^	Brune V, 2008	11 cases	isolated lymphoma cells	Affymetrix HG-U133 Plus 2.0 Array (GPL570)
GSE31312	Li Y, 2011	498 cases	lymphoma tissue	Affymetrix HG-U133 Plus 2.0 Array (GPL570)
GSE56315	Bødker JS, 2014	89 cases 33 controls	lymphoma tissue (case) isolated normal B cells (control)	Affymetrix HG-U133 Plus 2.0 Array (GPL570)
GSE64555	Linton K, 2014	40 cases	lymphoma tissue	Affymetrix HG-U133 Plus 2.0 Array (GPL570)
GSE69053	Sha C, 2015	212 cases	lymphoma tissue	Illumina HumanRef-8 WG-DASL v3.0 (GPL8432) Illumina HumanHT-12 WG-DASL V4.0 (GPL14951)
GSE86613	Bødker JS, 2016	41 cases	lymphoma tissue	Affymetrix HG-U133 Plus 2.0 Array (GPL570)
***Mantle cell lymphoma (MCL**)*				
GSE21452	Staudt LM, 2010	64 cases	lymph nodes	Affymetrix HG-U133 Plus 2.0 Array (GPL570)
GSE36000	Jares P, 2012	38 cases	isolated lymphoma cells	Affymetrix HG-U133 Plus 2.0 Array (GPL570)
GSE70910	Liu D, 2015	55 cases	lymph nodes, peripheral blood	Affymetrix HG-U133 Plus 2.0 Array (GPL570)
GSE93291	Staudt LM, 2017	59 cases	lymph nodes	Affymetrix HG-U133 Plus 2.0 Array (GPL570)

^1^The one control sample in this study was the mixed five control samples.

^2^This dataset included 17 HL samples, 11 DLBCL samples, and 25 controls. The number of control samples was shown in the HL group and was not repeated in the DLBCL group.

## DISCUSSION

Accurate and effective diagnosis is critical to the appropriate treatment of lymphoma. Although many new techniques are used for the diagnosis of lymphoma, such as immunohistochemical tests, flow cytometry, cytogenetic, and other molecular biology techniques [32], the most effective diagnostic strategy is still tissue biopsy [33]. Given that some genes show extremely high mutation frequencies in certain types of lymphoma, using a single-gene mutation or a combination of mutations may accurately diagnose few types of lymphomas [34]. However, the diagnosis of most lymphomas using genetic mutations may not achieve the desired accuracy. Previous transcriptome studies have revealed that there are a large number of abnormally expressed genes in different types of lymphomas compared with normal tissues [29, 35–37]. These highly differential genes may be used as diagnostic and prognostic markers for lymphomas [38]. The difference in clinical treatment and prognosis of B-cell-derived lymphoma is correlated to its molecular heterogeneity. In this study, lymphoma marker genes and specific diagnostic models are proposed, which are helpful to improve the diagnosis accuracy of HL, DLBCL, and MCL. These results indicate that there are certain differences at the molecular level among HL, DLBCL, and MCL, which provides some insights for the molecular diagnosis and prognosis assessment of these three types of lymphomas.

Gene expression profiling has broad application prospects in tumor diagnosis [39], and numerous novel biomarkers have been identified in the most common types of B-cell, T-cell, and NK-cell lymphomas [40]. Multiple diagnostic models based on the combined effects of tumor biomarkers have been developed and show high prediction accuracy. A previous report constructed two logistic regression models based on mammography features and demographic data; both of these models showed high accuracy for breast cancer diagnosis [41]. A logistic regression model integrating transcriptome and clinical data also showed high diagnostic accuracy in lung cancer [42]. Furthermore, using machine learning methods to construct tumor diagnostic models is also an effective strategy. Diagnostic models based on support vector machines and their derived methods by feature extraction of transcriptome data exhibited high prediction accuracy in multiple cancer datasets [43, 44]. The sample size is an important factor affecting the accuracy of the diagnostic model [45]. In this study, the sample size of DLBCL is relatively large, while the sample size of HL and MCL is relatively small, and the final multigene diagnosis models also showed the highest diagnostic accuracy for DLBCL. In future work, a larger sample size can be used to develop more accurate tumor-specific diagnosis or prognosis models. With the expansion of the sample size, it is expected to be further upgraded to a personalized prediction model.

The screened HL marker genes were mainly involved in actin- and cytoskeleton-related functions. Multiple studies showed that the actin cytoskeleton plays a crucial role in aging and apoptosis [46], and the dysfunction of the actin cytoskeleton correlated to many age-related diseases, such as cancer [47]. Actin polymerization and actin-myosin interactions directly drive the movement and migration of lymphocytes [48]. A proteomics study showed that several upregulated proteins were involved in the regulation of the cytoskeleton and/or cell migration in HL [49]. Inhibited cytoskeleton-related proteins promoted the differentiation of Hodgkin's and Reed-Sternberg (H/RS) cells toward terminal B-cells in HL cell lines [50]. DLBCL marker genes were mostly enriched in chromatin modification and regulation processes. Increased variations in chromatin modification were correlated with aging [51], studies showed that these epigenetic factors can also induce tumorigenesis [52]. Mutations in chromatin modification-related genes are correlated to gene expression profiles and clinical outcomes in DLBCL [53]. These genes can be used as signatures for evaluating the effect of medical treatments on DLBCL [54, 55]. Several immunoglobulin (Ig) subunit genes were chosen as MCL marker genes in this study. Alterations in IgG glycosylation patterns have been observed in aging and various cancers [56]. The regulation and modification of Ig are essential to maintain immune homeostasis *in vivo* [56]. The imbalanced Ig heavy and light chain stereotypy was found in MCL [57].

The above reports indicated that the marker genes screened in this study correlated with the specific biological changes of different lymphomas. Furthermore, these marker genes also showed high diagnostic accuracy in other tumors and correlated with tumorigenesis and prognosis. A previous study showed that MYH2 was correlated with multiple prognostic factors in lymph-node-negative primary breast cancer [58]. Downregulated IL26 promotes anaplastic large cell lymphoma cell growth and survival [59]. Gastric lipase (LIPF) is highly expressed in the normal stomach and showed significantly low expression in gastric adenocarcinoma, suggesting that it can be used as a diagnostic and prognostic indicator for gastric cancer [60, 61]. Low expression of PRB3 was found to be associated with tumor recurrence in prolactinomas [62] and salivary gland acinic cell carcinoma [63]. Besides, there are several representative markers associated with each subtype, such as CD15, CD30, CD45, and PD-L1 for HL [10], CD5, MYC, BCL2, and BCL6 for DLBCL [9], and CCND1, CD5, and SOX11 for MCL [7]. However, most of these genes did not meet the differential expression screening criteria. Therefore, these genes are not included in the specific markers screened in this study.

In conclusion, screening for tumor-specific biomarkers requires the rigorous consideration of differences between tumor and normal cells as well as the differences among different tumors or subtypes. The present study provides the transcriptome data-based reference markers, which may help the diagnosis of HL, DLBCL, and MCL when combined with other clinical markers. As there are multiple subtypes of lymphoma according to the WHO classification, whether the currently obtained marker genes can be used to diagnose other types of lymphomas requires further research. One potential shortcoming in this study is the sample size of different lymphoma datasets varies largely, especially for the control group is relatively low. The present study provides a molecular diagnostic method, a reference for tumor diagnosis with a subtle difference to clarify tumor subtypes.

## MATERIALS AND METHODS

### Lymphoma dataset collection

Transcriptome datasets of HL, DLBCL, and MCL were downloaded from the Gene Expression Omnibus (GEO) database (https://www.ncbi.nlm.nih.gov/geo/). The dataset selection criteria were as follows: (1) all datasets were genome-wide; (2) the number of samples in each dataset must be ≥ 10; (3) all samples were non-cell-line samples; and (4) complete microarray data (raw or normalized) were available. If a dataset contained any of the following items, it was excluded: (1) the number of samples was less than 3 for cases or controls; (2) the samples were treated with drugs or other agents; and (3) serious RNA degradation or the number of detected genes was too small. Based on the above criteria, 14 datasets were chosen for the integrated analysis (GSE12453, GSE13996, GSE17920, GSE21452, GSE31312, GSE36000, GSE47044, GSE56315, GSE64555, GSE69053, GSE70910, GSE7788, GSE86613 and GSE93291). The sample type of most data is lymphoma tissue, only a small part of the data derived from isolated lymphoma cells, the details of these datasets are provided in Table 1. In total, the collected datasets contained 240 HL samples, 891 DLBCL samples, 216 MCL samples, and 64 healthy samples.

### Data preprocessing

R statistical software v3.3.3 (https://www.r-project.org/) was used to perform data preprocessing. Because these datasets contain different microarray platforms, they were grouped into 15 batches according to the study and platform. Each batch contained only one study and one platform (Supplementary Table 4). Gene annotation, integration, and renormalization of the 15 batches were carried out using custom-designed Python code. The method and scripts are detailed in our previous publications [64, 65]. Because there were missing values for genes in a few samples, the mean expression value of these genes in the whole sample was used to replace the missing data. Fortunately, the missing values had little effect on the data (Supplementary Figure 7). After global renormalization, the distribution of gene expression values across all studies had a consistent range (Supplementary Figure 8). Heatmap in the pheatmap package in R was used to show all gene expression profiles in the integrated and the global renormalized datasets. The method of unsupervised clustering was chosen as "ward.D". There was a strong batch effect in the integrated datasets, and this batch effect has been mostly eliminated in the global renormalized datasets (Supplementary Figure 9).

### Differential expression analysis

Differential gene expression analysis was performed using the empirical Bayesian algorithm in the limma package in R [66]. Up- and downregulated genes were defined as a log2 transformed fold-change (logFC) ≥ 1 or ≤ -1 for lymphoma samples compared with controls. Because the difference between lymphoma groups was smaller than the difference between lymphoma samples and controls, the fold-change cutoff was set as 1.2. A false discovery rate (FDR)-corrected *P*-value ≤ 0.05 was considered significant.

### Screening of lymphoma marker genes

Lymphoma-specific genes were defined as the intersection of specific differentially expressed genes in lymphoma samples compared to controls and the common intergroup differentially expressed genes in different lymphoma groups. For example, the filtered HL-specific genes are differentially expressed between HL vs. control with no difference in DLBCL vs. control or MCL vs. control and are differentially expressed between HL vs. DLBCL and HL vs. MCL. To ensure that the screened marker genes have a relatively large differential expression compared to other types of lymphoma, the lymphoma marker genes were defined as lymphoma-specific genes with a mean absolute value of intergroup fold-change ≥ 0.5.

### Gene-gene interaction analysis

Considering that the gene-gene interactions between the screened marker genes and other genes may affect prediction accuracy, Pearson correlation analysis was used to calculate the correlation coefficient between each marker gene and all other genes. An FDR corrected *P*-value ≤ 0.05 was considered significantly correlated. A multiple logistic regression model was used to analyze the effect of each marker gene on the corresponding lymphoma. The top 10 significantly correlated genes (filtered by significance) were used as covariates for model correction.

### GO enrichment analysis

The information on human genes and related GO biological functions were downloaded from the QuickGO database (http://www.ebi.ac.uk/QuickGO-Beta/). GO enrichment analysis was performed using a hypergeometric test and the formula shown in a previous report [67]. An FDR corrected *P*-value ≤ 0.05 was considered significantly enriched.

### Functional interaction analysis

The GeneMANIA application [68] in Cytoscape v3.4.0 was used to perform functional interaction analysis of marker genes in three types of lymphoma. The interaction networks were built with the default parameter settings. The application predicts 20 query genes that are correlated to the input genes and generate a functional association network based on their relationships. The functional enrichment results of the genes in the network were automatically generated, and an FDR-corrected *P*-value ≤ 0.05 was considered significantly enriched.

### Prognostic analysis

Two datasets (GSE17920 and GSE31312) had prognostic information. The GSE17920 dataset (HL) contains multiple prognostic indicators but no survival data, and the GSE31312 dataset (DLBCL) contains complete overall and progression-free survival information. The difference in HL marker genes regarding prognostic indicators was determined using Student’s *t*-test. Survival analysis was conducted using the survival package in R. The effects of DLBCL marker genes on overall and progression-free survival were assessed using Kaplan–Meier survival curves.

### Single-gene and multigene prediction models

The single-gene prediction model and the multigene prediction model were built using the lymphoma marker genes. A univariate logistic regression model was used to calculate the odds ratios of the screened lymphoma marker genes in each type of lymphoma. For the single-gene prediction models, the specified type of lymphoma was classified as "case", whereas the healthy samples and the other two types of lymphomas were classified as "control". The receiver operating characteristic (ROC) curve and the area under the curve (AUC) of the single marker genes were calculated using the pROC package in R. The model with the largest AUC was defined as the optimal model. A stepwise modeling strategy was used to screen the optimal multigene combination models for each type of lymphoma. First, a gene with the largest AUC was selected. Then, we used a multivariate logistic regression model to generate the combined effect of the selected gene and each of the remaining genes. Next, we selected the best two-gene model with the highest AUC and repeated the previous steps. Finally, we selected the optimal model with the highest AUC in each multigene combination model.

## Supplementary Materials

Supplementary Figures

Supplementary Tables
