# Supplementary material for: Transcriptome integration analysis and specific diagnosis model construction for Hodgkin's lymphoma, diffuse large B-cell lymphoma, and mantle cell lymphoma
Source: Aging (Albany NY). 2021 Apr 22;13(8):11833–59. doi: 10.18632/aging.202882 (PMC8109084; doi:10.18632/aging.202882)
Supplement: Supplementary Figures [file aging-13-202882-s001.pdf]

SUPPLEMENTARY FIGURES

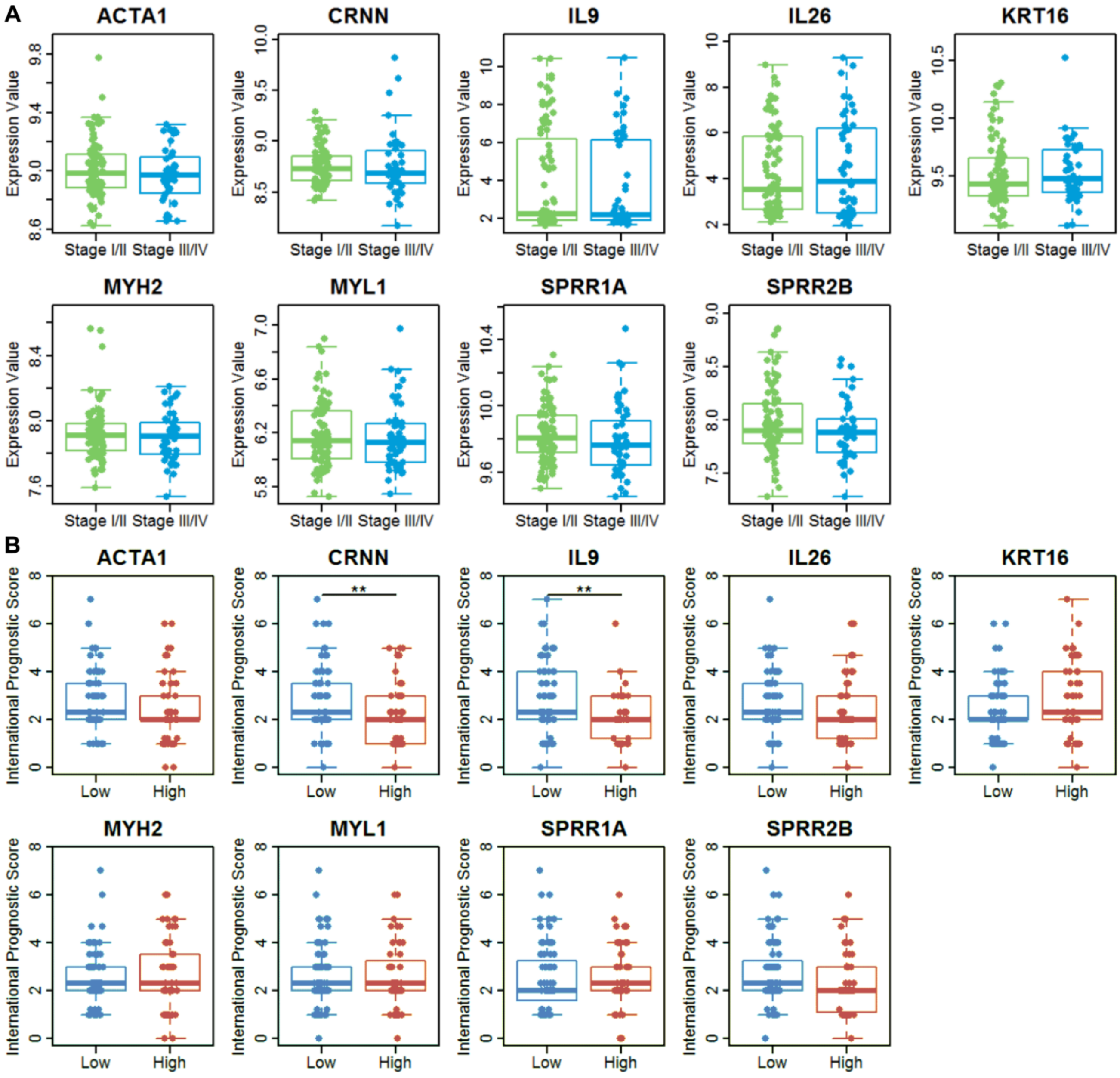

Supplementary Figure 1. Correlation of HL marker genes and prognostic indicators. (**A**) Correlation between HL marker genes and stage. (**B**) Correlation between HL marker genes and International Prognostic Score. The genes were divided into high and low groups based on the median expression value. HL, Hodgkin's lymphoma. Significance: $^{*}P < 0.05$, $^{**}P < 0.01$, $^{***}P < 0.001$.

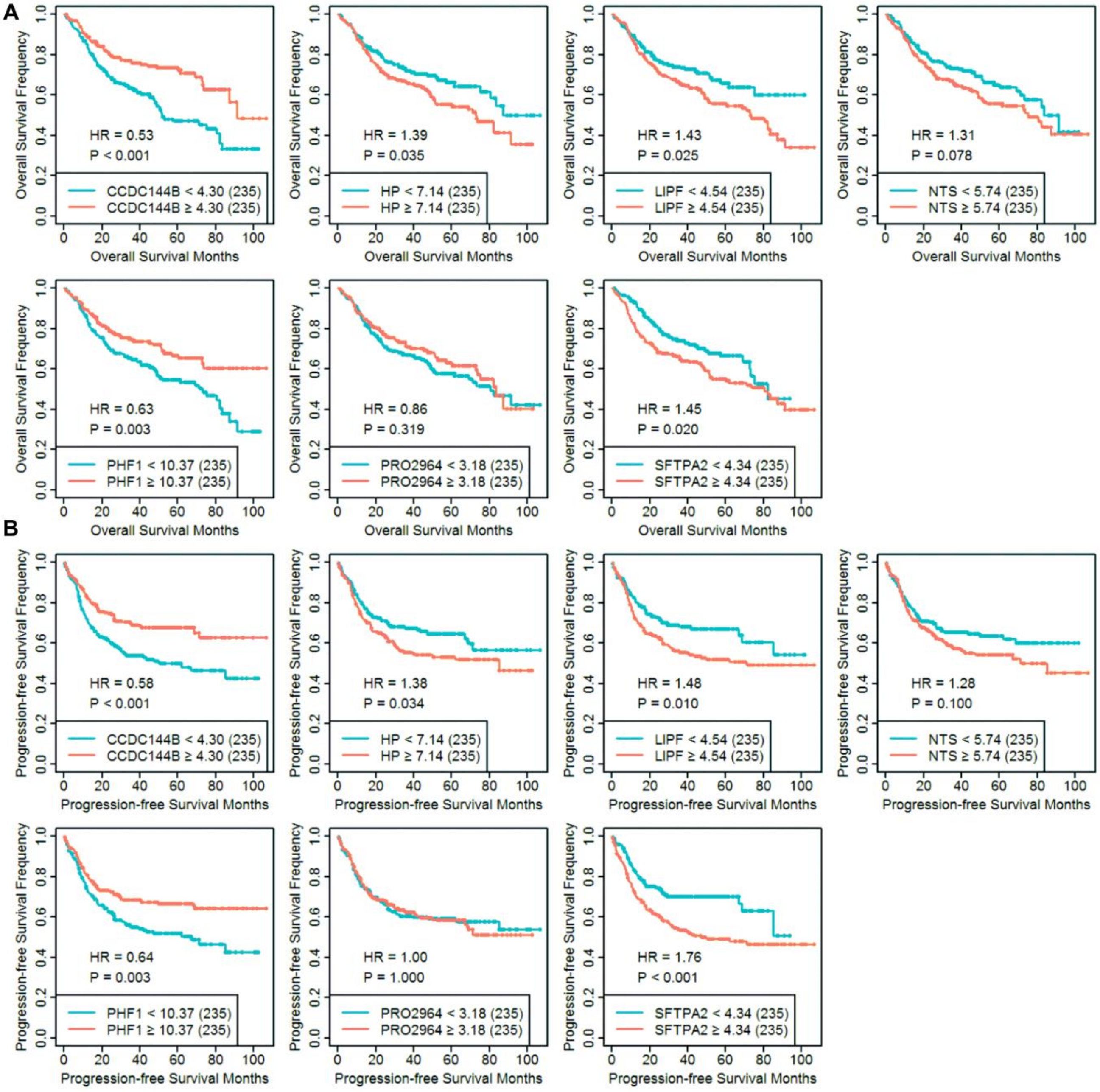

Supplementary Figure 2. Effect of DLBCL marker genes on patient overall survival (**A**) and progression-free survival (**B**). The genes were divided into high and low groups based on the median expression value.

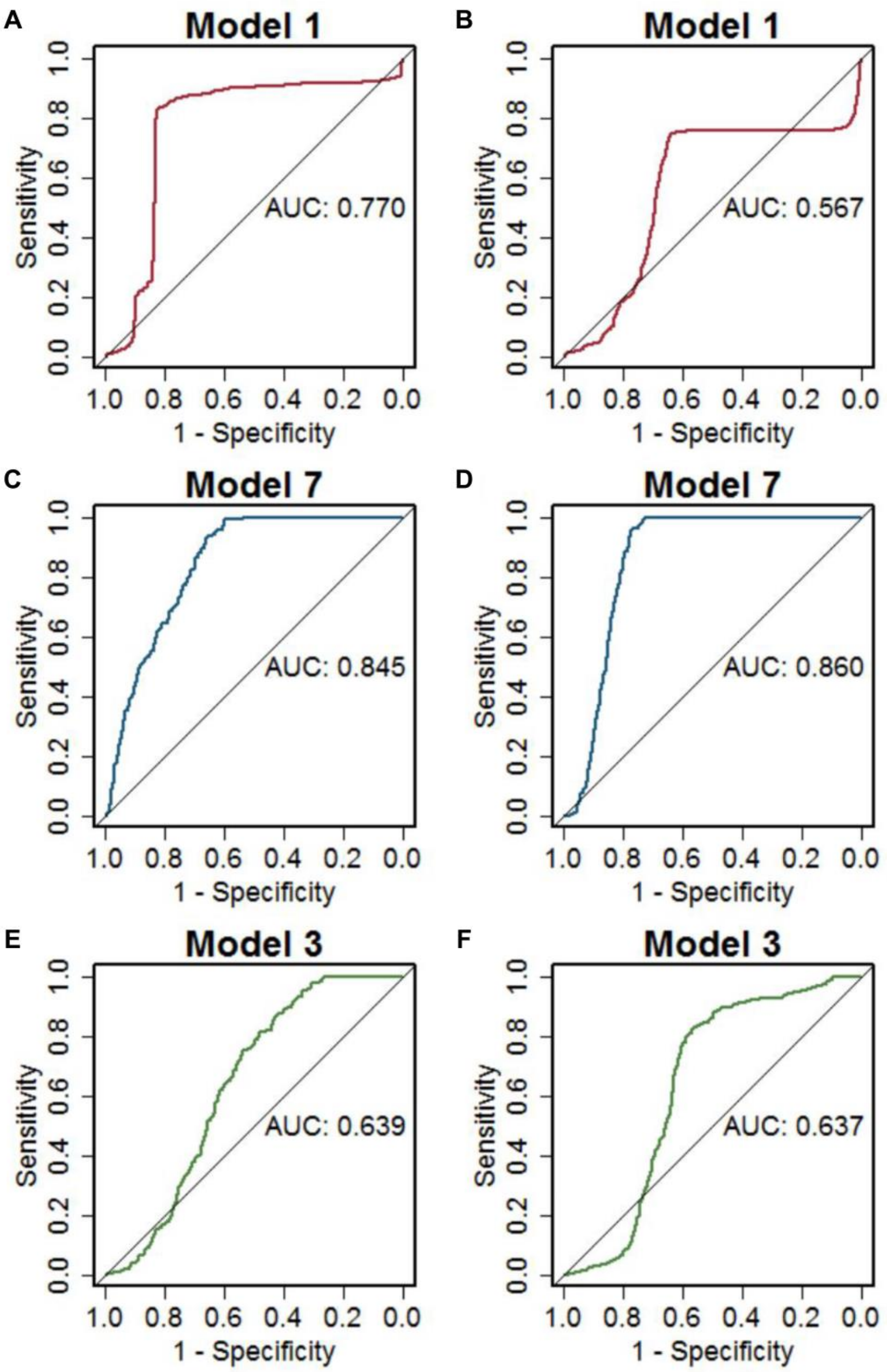

Supplementary Figure 3. The marker genes showed relatively poor specificity for the other two types of lymphomas (corresponding to the optimal model in Figure 4). (**A**) The classification performance of HL marker genes on DLBCL. (**B**) The classification performance of HL marker genes on MCL. (**C**) The classification performance of DLBCL marker genes on HL. (**D**) The classification performance of DLBCL marker genes on MCL. (**E**) The classification performance of MCL marker genes on HL. (**F**) The classification performance of MCL marker genes on DLBCL. HL, Hodgkin's lymphoma; DLBCL, diffuse large B-cell lymphoma; MCL, mantle cell lymphoma.

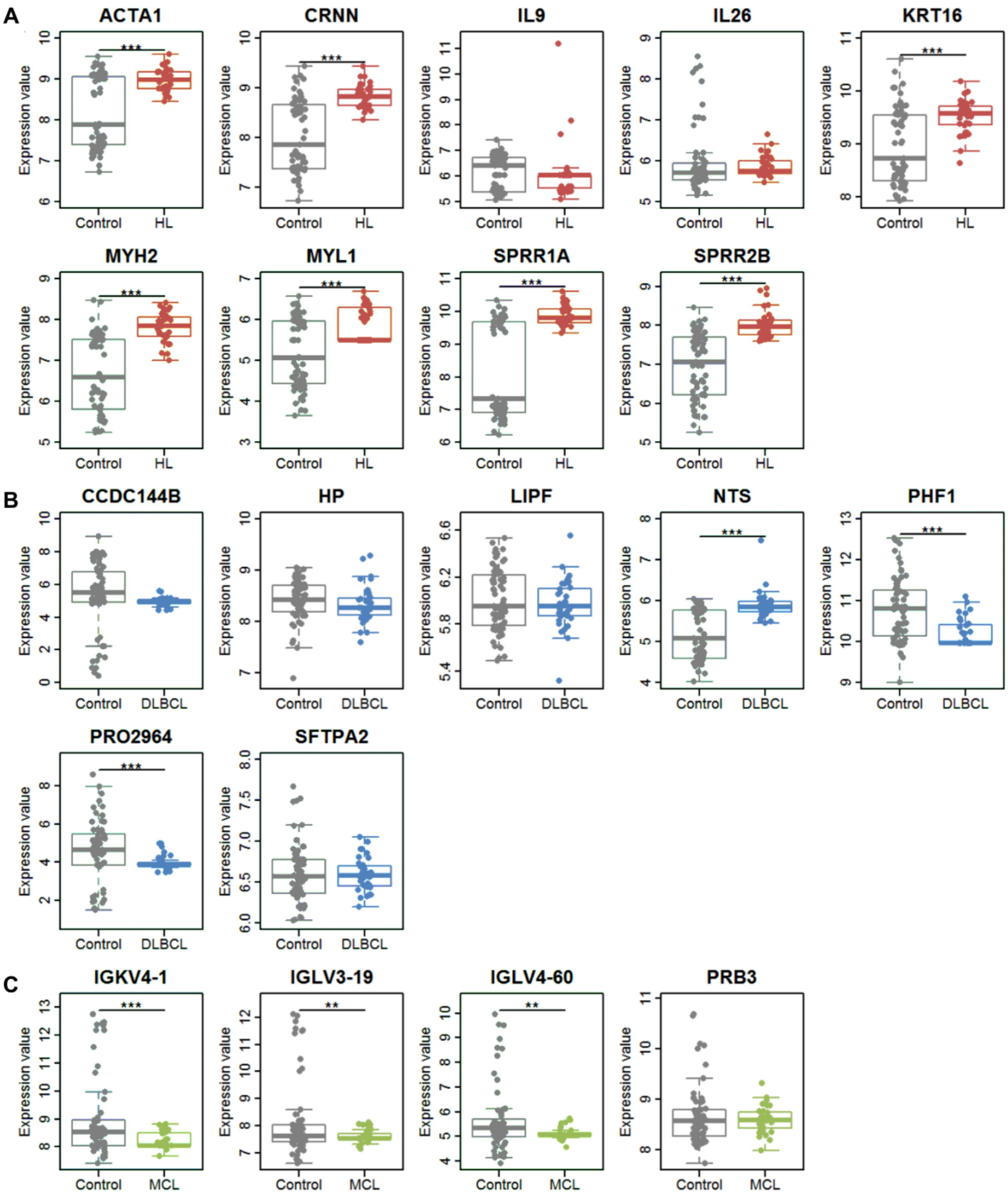

Supplementary Figure 4. Expression of lymphoma marker genes in the data derived from isolated lymphoma cells and normal B cells. The corresponding datasets see in Table 1. (**A**) Expression of HL marker genes between HL samples and controls. (**B**) Expression of DLBCL marker genes between DLBCL samples and controls. (**C**) Expression of MCL marker genes between MCL samples and controls. HL, Hodgkin's lymphoma; DLBCL, diffuse large B-cell lymphoma; MCL, mantle cell lymphoma. Significance: $^{*}P < 0.05$, $^{**}P < 0.01$, $^{***}P < 0.001$.

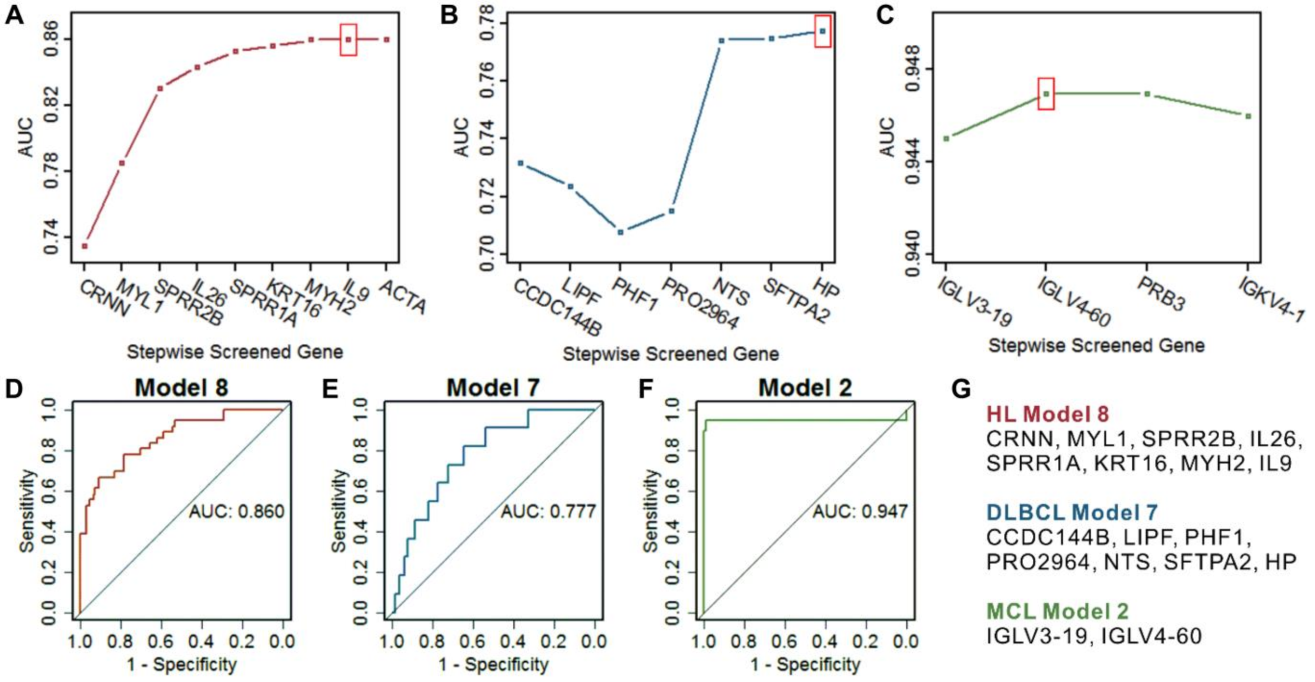

Supplementary Figure 5. Screening of the optimal multigene prediction model for three lymphomas using the data derived from isolated lymphoma cells. The corresponding datasets see in Table 1. (**A**–**C**) Stepwise screened multigene prediction models in HL, DLBCL and MCL. From left to right on the x-axis (stepwise screened genes), each additional gene corresponds to a model [for example, in (A), CRNN represents model 1, which contains one gene of CRNN, MYL1 represents model 2, which contains two genes including CRNN and MYL1]. The red box shows the optimal model for each type of lymphoma. (**D**–**F**) ROC curves of the screened optimal models for each type of lymphoma. (**G**) Genes in the screened optimal models for three lymphomas. HL, Hodgkin's lymphoma; DLBCL, diffuse large B-cell lymphoma; MCL, mantle cell lymphoma.

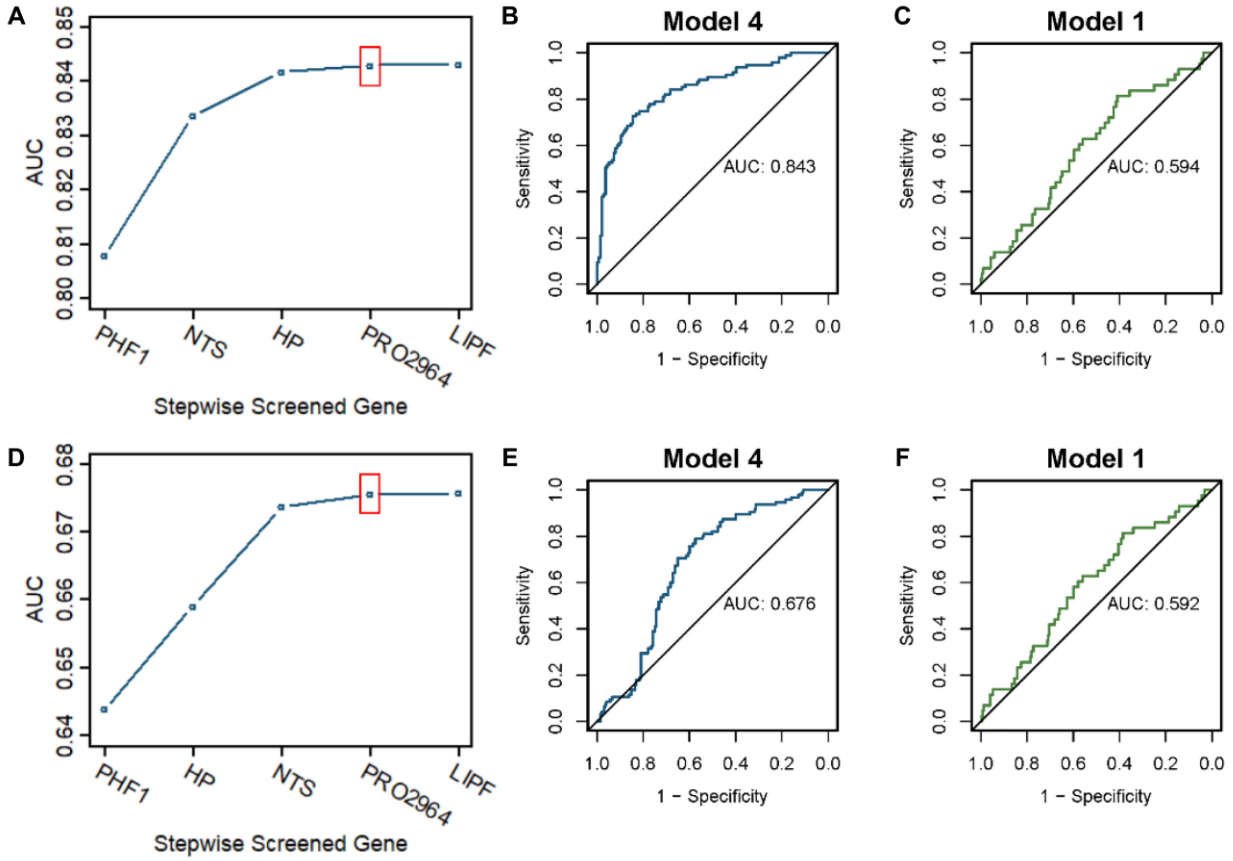

Supplementary Figure 6. The prediction performance of the lymphoma marker genes in the validation dataset of GSE132929. The dataset including Burkitt's lymphoma (BURK), diffuse large B-cell lymphoma (DLBCL), double hit lymphoma (DHL), follicular lymphoma (FL), mantle cell lymphoma (MCL), medial zone lymphoma (MZL), and other high-grade B-cell lymphomas (no Hodgkin's lymphoma (HL) or controls). There were only 5 DLBCL marker genes and 1 MCL marker gene (PRB3) in the GSE132929 dataset. (**A**–**C**) The validation dataset does not include BURK. (**D**–**F**) The validation dataset includes BURK. (**A**, **D**) Stepwise screened multigene prediction models in DLBCL. From left to right on the x-axis (stepwise screened genes), each additional gene corresponds to a model [for example, in (A), PHF1 represents model 1, which contains one gene of PHF1, NTS represents model 2, which contains two genes including PHF1 and NTS]. The red box shows the optimal model for each type of lymphoma. (**B**, **E**) ROC curves of the screened optimal model for DLBCL. (**C**, **F**) ROC curves of the PRB3 model for MCL.

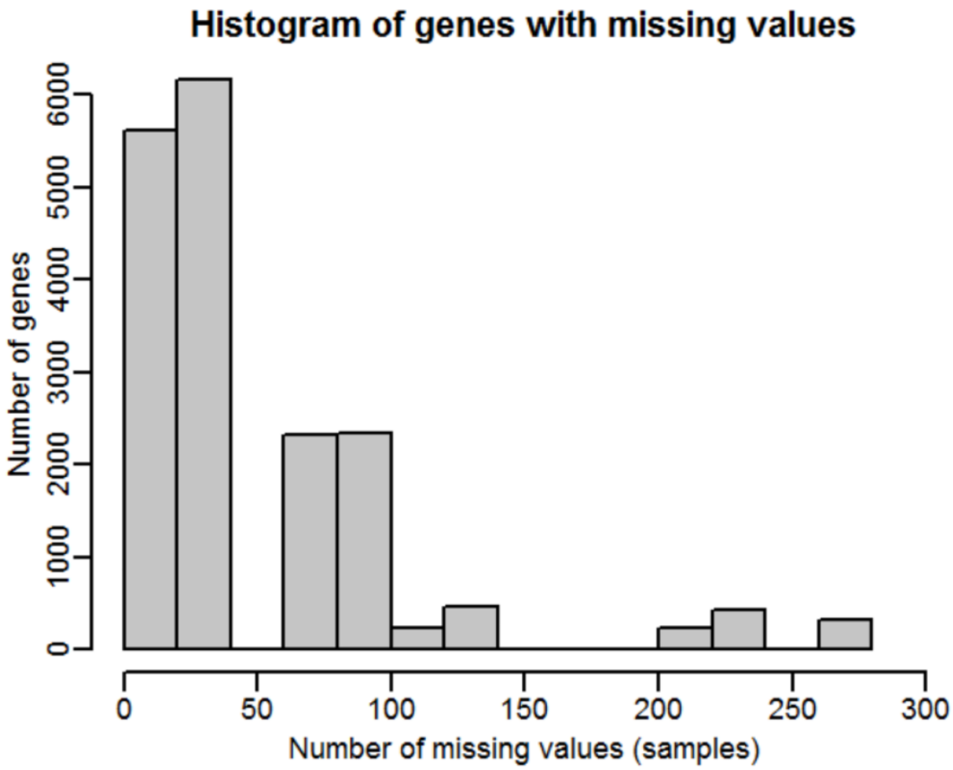

Supplementary Figure 7. Histogram of genes with missing values. The x-coordinate indicates how many samples have missing values. This study collected 1411 samples, and the total number of genes was 18116. The figure shows that most of the samples' expression data are relatively complete (80% of samples have no missing values, 13% of samples have less than 3% missing values, and only 7% of samples have more than 10% missing values).

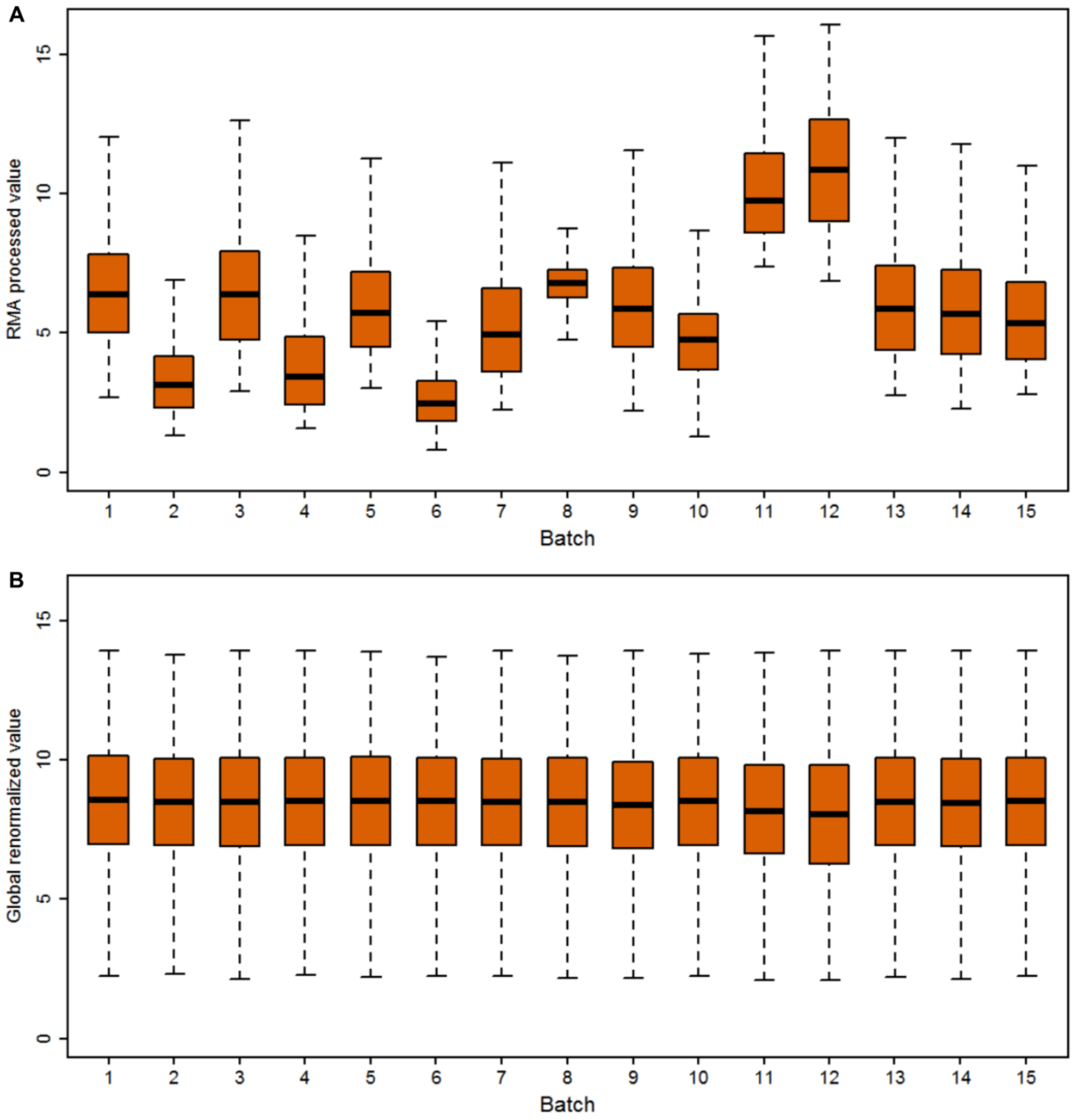

Supplementary Figure 8. The distribution of the RMA-processed gene expression values (**A**) and the global renormalized gene expression values (**B**) of the lymphoma datasets. Details of these batches see Supplementary Table 4. There was a relatively large deviation in the distribution of gene expression values across these batches in the RMA-processed gene expression values. The distribution of gene expression values across these batches had a consistent range in the global renormalized gene expression values.

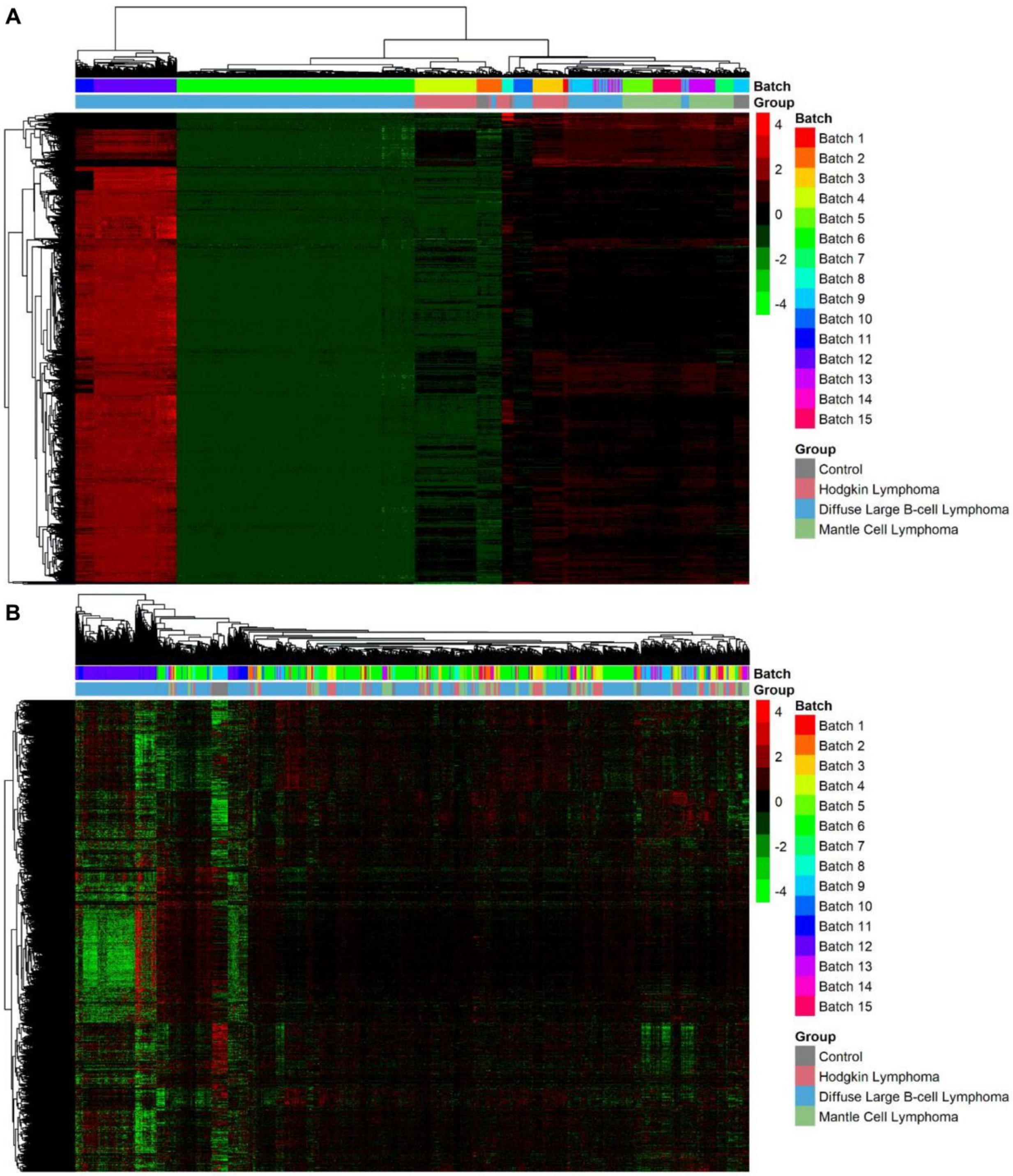

Supplementary Figure 9. Heatmap of the gene expression profiles in the integrated (**A**) and the global renormalized (**B**) lymphoma datasets. All gene expression values were z-score converted. There was a strong batch effect in the integrated datasets whereas only a weak batch effect in the global renormalized datasets.
