# Supplementary material for: Transcriptome integration analysis and specific diagnosis model construction for Hodgkin's lymphoma, diffuse large B-cell lymphoma, and mantle cell lymphoma
Source: Aging (Albany NY). 2021 Apr 22;13(8):11833–59. doi: 10.18632/aging.202882 (PMC8109084; doi:10.18632/aging.202882)
Supplement: Supplementary Tables [file aging-13-202882-s002.pdf]

SUPPLEMENTARY TABLES

Supplementary Table 1. Enriched GO biological processes of lymphoma specific genes.[1]

ID	Term	FDR	Genes in GOBP
Hodgkin's lymphoma			
GO:0070268	cornification	7.43E–08	KRT24, SPRR2B, SPRR1A, KRT16, PI3
GO:0018149	peptide cross-linking	2.71E–06	SPRR2B, SPRR1A, PI3
GO:0006936	muscle contraction	4.10E–06	MYH2, ACTA1, MYL1, MYOT
GO:0030049	muscle filament sliding	5.09E–06	MYH2, ACTA1, MYL1
GO:0030216	keratinocyte differentiation	1.15E–05	SPRR2B, SPRR1A, KRT16
GO:0008544	epidermis development	2.00E–05	SPRR2B, SPRR1A, KRT16
GO:2000648	positive regulation of stem cell proliferation	2.64E–04	SOX11
GO:0071305	cellular response to vitamin D	2.64E–04	PHEX
GO:0010226	response to lithium ion	2.64E–04	ACTA1
GO:0060174	limb bud formation	3.16E–04	SOX11
Diffuse large B-cell lymphoma			
GO:0021846	cell proliferation in forebrain	1.24E–05	FGF8, LHX5
GO:0007512	adult heart development	3.71E–05	MYH6, MYH7
GO:0014898	cardiac muscle hypertrophy in response to stress	4.62E–05	MYH6, MYH7
GO:0019226	transmission of nerve impulse	6.86E–05	CACNG7, CNTNAP2
GO:2000311	regulation of AMPA receptor activity	9.70E–05	CACNG7, SHANK1
GO:0071625	vocalization behavior	9.70E–05	CNTNAP2, SHANK1
GO:0002026	regulation of the force of heart contraction	1.14E–04	MYH6, MYH7
GO:0035176	social behavior	1.38E–04	CNTNAP2, TH, SHANK1
GO:0006941	striated muscle contraction	1.53E–04	MYH6, MYH7
GO:0006936	muscle contraction	1.68E–04	MYH13, GALR2, MYH6, MYH7
Mantle cell lymphoma			
GO:0002377	immunoglobulin production	4.62E–07	IGLV4-60, IGKV4-1
GO:0006956	complement activation	3.92E–06	IGKV4-1, IGLV3-19
GO:0006958	complement activation, classical pathway	6.29E–06	IGKV4-1, IGLV3-19
GO:0030449	regulation of complement activation	7.87E–06	IGKV4-1, IGLV3-19
GO:0038096	Fc-gamma receptor signaling pathway involved in phagocytosis	1.75E–05	IGKV4-1, IGLV3-19
GO:0048333	mesodermal cell differentiation	3.59E–05	INHBA
GO:0071372	cellular response to follicle-stimulating hormone stimulus	3.59E–05	INHBA
GO:2001241	positive regulation of extrinsic apoptotic signaling pathway in absence of ligand	4.13E–05	INHBA
GO:0061029	eyelid development in camera-type eye	4.70E–05	INHBA
GO:0071397	cellular response to cholesterol	5.28E–05	INHBA

[1]All GO biological process enrichment results are sorted by FDR and showed the top 10 enriched results for each lymphoma.

Supplementary Table 2. Enriched GO biological processes of lymphoma marker genes.[1]

ID	Term	FDR	Genes in GOBP
Hodgkin's lymphoma			
GO:0045407	positive regulation of interleukin-5 biosynthetic process	<1.00E–16	IL9
GO:0030049	muscle filament sliding	1.77E–07	ACTA1, MYH2, MYL1
GO:0030216	keratinocyte differentiation	4.02E–07	KRT16, SPRR1A, SPRR2B
GO:0008544	epidermis development	9.04E–07	KRT16, SPRR1A, SPRR2B
GO:0070268	cornification	2.52E–06	KRT16, SPRR1A, SPRR2B
GO:0006936	muscle contraction	2.56E–06	ACTA1, MYH2, MYL1
GO:0043503	skeletal muscle fiber adaptation	2.56E–06	ACTA1
GO:0009991	response to extracellular stimulus	6.71E–06	ACTA1
GO:0018149	peptide cross-linking	6.71E–06	SPRR1A, SPRR2B
GO:0071417	cellular response to organonitrogen compound	1.17E–05	ACTA1
Diffuse large B-cell lymphoma			
GO:2000296	negative regulation of hydrogen peroxide catabolic process	1.71E–06	HP
GO:0061086	negative regulation of histone H3-K27 methylation	6.15E–06	PHF1
GO:0061087	positive regulation of histone H3-K27 methylation	7.32E–06	PHF1
GO:0051354	negative regulation of oxidoreductase activity	7.32E–06	HP
GO:0050880	regulation of blood vessel size	2.30E–05	NTS
GO:0006108	malate metabolic process	2.56E–05	LIPF
GO:0045814	negative regulation of gene expression, epigenetic	1.19E–04	PHF1
GO:0007585	respiratory gaseous exchange	1.32E–04	SFTPA2
GO:0006953	acute-phase response	1.42E–04	HP
GO:0010942	positive regulation of cell death	1.61E–04	HP
Mantle cell lymphoma			
GO:0002377	immunoglobulin production	2.49E–08	IGKV4-1, IGLV4-60
GO:0006956	complement activation	3.85E–07	IGKV4-1, IGLV3-19
GO:0006958	complement activation, classical pathway	5.55E–07	IGKV4-1, IGLV3-19
GO:0006955	immune response	6.35E–07	IGKV4-1, IGLV3-19, IGLV4-60
GO:0030449	regulation of complement activation	6.35E–07	IGKV4-1, IGLV3-19
GO:0038096	Fc-gamma receptor signaling pathway involved in phagocytosis	1.43E–06	IGKV4-1, IGLV3-19
GO:0050776	regulation of immune response	5.29E–06	IGKV4-1, IGLV3-19
GO:0038095	Fc-epsilon receptor signaling pathway	5.29E–06	IGKV4-1, IGLV3-19
GO:0050900	leukocyte migration	7.66E–06	IGKV4-1, IGLV3-19
GO:0006898	receptor-mediated endocytosis	8.45E–06	IGKV4-1, IGLV3-19

[1]All GO biological process enrichment results are sorted by FDR and showed the top 10 enriched results for each lymphoma.

Supplementary Table 3. Logistic regression between marker genes and lymphomas.

Variable	Crude		Adjusted[1]		Adjusted top 10 significantly correlated genes[1]
	OR (95% CI)	*P*	OR (95% CI)	*P*	
Hodgkin's Lymphoma					
MYH2	1.99 (1.75–2.27)	< 0.001	2.55 (1.97–3.29)	< 0.001	MYL1, ACTA1, KBTBD10, MB, CKM, MYH1, SMPX, CSRP3, MYBPC1, ASB5
SPRR2B	1.77 (1.59–1.98)	< 0.001	1.25 (0.97–1.60)	0.084	SPRR1A, S100A7, SPRR1B, SPRR3, KRT6A, S100A2, KRT6B, CLCA2, CRCT1, CRNN
MYL1	1.89 (1.65–2.18)	< 0.001	1.43 (1.00–2.04)	0.048	KBTBD10, MB, MYBPC1, SMPX, MYH1, MYH2, MYOT, CSRP3, TNNT1, MYL2
SPRR1A	1.61 (1.48–1.74)	< 0.001	1.96 (1.26–3.05)	0.003	KRT13, KRT6A, KRT6B, S100A7, SPRR1B, SPRR2B, SPRR3, S100A2, SPINK5, KRT14
CRNN	1.68 (1.47–1.92)	< 0.001	1.95 (1.41–2.69)	< 0.001	CLCA2, DSG3, TGM3, KRT6B, CRCT1, SPINK7, KRT16, RHCG, TMPRSS11D, KRT78
ACTA1	1.71 (1.50–1.95)	< 0.001	1.51 (1.13–2.03)	0.006	CKM, MYH2, MYL2, CA3, MYH1, MYLPF, TNNC1, TNNC2, MYH7, XIRP2
IL9	0.39 (0.35–0.44)	< 0.001	0.40 (0.33–0.48)	< 0.001	IL13RA2, IL26, AMPH, DHRS2, CCL11, CLC, TFPI2, MFAP2, CYP4Z1, SCG2
KRT16	1.51 (1.35–1.69)	< 0.001	1.78 (1.31–2.43)	< 0.001	KRT6B, LY6D, DSG3, CLCA2, CRNN, RHCG, TGM3, KRT14, SERPINB2, KRT17
IL26	0.39 (0.33–0.47)	< 0.001	1.11 (0.87–1.41)	0.404	IL9, CYP4Z1, CLC, IL13RA2, IL22, DHRS2, COL6A6, CCL26, AMPH, CCL23
Diffuse Large B-cell Lymphoma					
LIPF	0.08 (0.06–0.11)	< 0.001	0.38 (0.25–0.60)	< 0.001	GKN1, CTSE, SFTPA2, OLFM4, DEFA6, KRT20, TMEM183A, MUC7, GKN2, RBP2
SFTPA2	0.20 (0.17–0.24)	< 0.001	0.21 (0.16–0.29)	< 0.001	LIPF, CTSE, GKN1, SLC34A2, TMEM183A, KRT4, MUC7, DEFA6, OLFM4, KRT20
CCDC144 B	0.48 (0.41–0.55)	< 0.001	0.46 (0.38–0.54)	< 0.001	TAF13, LOC100287927, ZNF90, GTF2I, PTENP1, PKD2L2, UGGT1, HIST1H4E, PTMS, LOC100509761
HP	0.31 (0.27–0.37)	< 0.001	0.36 (0.30–0.43)	< 0.001	ALB, FGA, AHSG, FGL1, PRG4, FGB, TTR, GC, FGG, C4BPA
PRO2964	0.53 (0.47–0.59)	< 0.001	0.65 (0.57–0.73)	< 0.001	HIST1H2BM, PTENP1, RAB30, HIST1H4B, ZNF814, ST6GAL1, HIST2H2AA3, HIST1H3I, SKIL, LOC100129112
NTS	1.94 (1.70–2.21)	< 0.001	1.85 (1.56–2.19)	< 0.001	CLEC4M, NPY1R, MMRN1, CCL20, MATN2, CETP, CCL21, TFPI, TSPAN7, SDPR
PHF1	0.64 (0.58–0.70)	< 0.001	0.63 (0.58–0.70)	< 0.001	RPSA, RPLP0, RPL35, RPL30, RPS12, RPL3, COX6B1, RPS29, RPLP1, RPS15
Mantle Cell Lymphoma					
IGLV3-19	0.48 (0.43–0.53)	< 0.001	0.16 (0.12–0.20)	< 0.001	IGLJ3, IGLV4-60, IGLV2-23, IGLV1-40, IGKV3-20, IGKV1-5, LOC100508797, IGKV1D-8, LOC100287927, IGLV6-57
IGKV4-1	0.58 (0.53–0.63)	< 0.001	0.29 (0.23–0.37)	< 0.001	IGKV1-5, IGLV2-23, IGLJ3, IGKV3-20, IGLV1-40, IGKC, SDC1, IGKV1D-8, IGLV3-19, IGLV4-60
PRB3	0.55 (0.49–0.61)	< 0.001	0.19 (0.13–0.26)	< 0.001	PRB4, PRB1, C20orf70, HTN1, CRISP3, AZGP1, LPO, CST5, PIP, TCN1
IGLV4-60	0.46 (0.40–0.53)	< 0.001	0.28 (0.20–0.39)	< 0.001	IGLV1-40, IGKV3-20, IGLJ3, LOC100287927, IGKV1-5, SDC1, IGLV3-19, IGLV6-57, IGKV1D-8, IGLV2-23

[1]Pearson correlation analysis was used to calculate the correlation between marker genes all other genes in the whole samples. A false discovery rate (FDR) corrected *P*-value ≤ 0.05 was considered as significant correlated. The top 10 significant correlated based on FDR *P*-value were used for covariates correction in logistic regression analysis.

Supplementary Table 4. Grouping the lymphoma datasets for data integration and global renormalization.

GEO ID	Category	Platform	Batch
GSE7788	HL, Control	GPL570	1
GSE12453	HL, DLBCL, Control	GPL570	2
GSE13996	HL	GPL571	3
GSE17920	HL	GPL570	4
GSE21452	MCL	GPL570	5
GSE31312	DLBCL	GPL570	6
GSE36000	MCL	GPL570	7
GSE47044	HL, Control	GPL6244	8
GSE56315	DLBCL, Control	GPL570	9
GSE64555	DLBCL	GPL570	10
GSE69053	DLBCL	GPL8432	11
GSE69053	DLBCL	GPL14951	12
GSE70910	MCL	GPL570	13
GSE86613	DLBCL	GPL570	14
GSE93291	MCL	GPL570	15

Abbreviations: HL, Hodgkin's lymphoma; DLBCL, diffuse large B-cell lymphoma; MCL, mantle cell lymphoma.
